# Characteristics of Dysphagia in Infants with Microcephaly Caused by Congenital Zika Virus Infection, Brazil, 2015

**DOI:** 10.3201/eid2308.170354

**Published:** 2017-08

**Authors:** Mariana C. Leal, Vanessa van der Linden, Thiago P. Bezerra, Luciana de Valois, Adriana C.G. Borges, Margarida M.C. Antunes, Kátia G. Brandt, Catharina X. Moura, Laura C. Rodrigues, Coeli R. Ximenes

**Affiliations:** Hospital Agamenon Magalhães, Recife, Brazil (M.C. Leal);; Universidade Federal de Pernambuco, Recife (M.C. Leal, T.P. Bezerra, M.M.C. Antunes, K.G. Brandt, C.R. Ximenes);; Hospital Barão de Lucena, Recife (V. van der Linden);; Association for Assistance of Disabled Children, Recife (V. van der Linden, L. de Valois);; Prived Clinic, Recife (A.C.G. Borges);; Real Hospital Português, Recife (C.X. Moura);; London School of Hygiene and Tropical Medicine, London, UK (L.C. Rodrigues)

**Keywords:** dysphagia, oral motor dysfunction, oral phase, pharyngeal phase, esophageal phase, aspiration risk, swallowing, infants, Zika virus infection, microcephaly, neurologic disease, congenital infection, congenital Zika syndrome, viruses, vector-borne infections, Brazil

## Abstract

We summarize the characteristics of dysphagia in 9 infants in Brazil with microcephaly caused by congenital Zika virus infection. The Schedule for Oral Motor Assessment, fiberoptic endoscopic evaluation of swallowing, and the videofluoroscopic swallowing study were used as noninstrumental and instrumental assessments. All infants had a degree of neurologic damage and showed abnormalities in the oral phase. Of the 9 infants, 8 lacked oral and upper respiratory tract sensitivity, leading to delays in initiation of the pharyngeal phase of swallowing. Those delays, combined with marked oral dysfunction, increased the risk for aspiration of food, particularly liquid foods. Dysphagia resulting from congenital Zika virus syndrome microcephaly can develop in infants >3 months of age and is severe.

## Abstract 2

Oral motor dysfunction begins after 3 months of age and is severe.

The identification of a congenital Zika syndrome (CZS) during the November 2015 microcephaly epidemic in Brazil raised many questions (*1*). So far, the main phenotypic indicator of CZS is microcephaly, but the full spectrum of CZS has not been described and will probably include other systemic abnormalities, and it may be shown that microcephaly is not present in all cases (*2*,*3*). Early research indicated that Zika virus has tropism for glial cells and neurons, a finding consistent with the severity of abnormalities in seen the fetal brain (*4*).

Swallowing is a complex sensorimotor process that depends on information from multiple levels of the central and peripheral nervous system. Swallowing occurs when descending excitatory and inhibitory signals from the cortex and subcortex and ascending signals from the oropharyngeal area trigger the central pattern generator in the bulbar reticular formation (*5*). The execution of the sensorimotor aspects associated with swallowing relies on functionally connected pathways between extrapyramidal cortical motor planning regions, centers controlling the brainstem and cranial nerves, and lower motor neurons. The swallowing process has 3 phases: oral, pharyngeal, and esophageal. Dysphagia can result from problems in any, of the 3 phases or in more than 1 phase (*6*). The oral phase and the initiation of the pharyngeal phase are under voluntary neural control, whereas the completion of the pharyngeal phase and the entire esophageal phase are under involuntary neural control (*7*).

There were a few early initial reports of onset of clinically diagnosed dysphagia in the first months of life of children with CZS; the most relevant are a case series about CZS with arthrogryposis in 7 infants (*8*) and another about CZS without microcephaly at birth in 13 infants (*2*). In the latter case series, 76% (10/13) of the infants had dysphagia, which was present in children with neurologic manifestations that were not very severe (*2*). Feeding problems in persons with neurologic diseases are mainly explained by brain damage leading to lack of swallowing coordination; abnormalities of posture; and abnormalities of digestive tract motility, such as gastroparesis and gastroesophageal reflux (*9*). Although these problems may be the cause of dysphagia seen in infants with CZS, we suggest that CZS-associated dysphagia might also be caused by anomalies of orofacial anatomy, oral and upper respiratory tract sensitivity, and changes in the motor function of the upper digestive tube caused primarily or secondarily by direct action of the virus. We undertook instrumental evaluation of dysphagia in children with CZS with the objective of describing the characteristics of this condition.

## Methods

### Study Design and Population

We conducted a descriptive, retrospective, case series study by reviewing the medical records of 9 children in Brazil with dysphagia and CZS microcephaly diagnosed during the 2015 epidemic of microcephaly. The study was conducted in 3 tertiary care institutions in Recife, Pernambuco State, Brazil: 1) Associação de Assistência à Criança Deficiente, the reference center for disabled children, where the infants with dysphagia were followed up by a neurologist and speech pathologist; 2) the Pediatric Otolaryngology and Pediatric Gastroenterology Outpatient Unit at the Hospital das Clínicas da Universidade Federal de Pernambuco, where infants received a complete dysphagia assessment, including fiberoptic endoscopic evaluation of swallowing (FEES); and 3) the Division of Radiology at Real Hospital Português, where infants underwent the videofluoroscopic swallowing study (VFSS).

The 9 infants were referred for dysphagia evaluation at 1 of the 3 study sites, where they underwent a complete instrumental evaluation. All infants met the following 3 criteria: 1) they had been diagnosed with microcephaly and had brain imaging results suggestive of congenital Zika virus infection; 2) other causes of infectious congenital microcephaly (toxoplasmosis, cytomegalovirus, rubella, syphilis, and HIV) had been excluded; and 3) their cerebrospinal fluid was Zika virus IgM–positive by capture ELISA (*10*). Microcephaly was defined as head circumference 2 SDs below the median for newborn gestational age and sex (*11*). Clinical neurologic evaluation included brain imaging, without contrast, by computed tomography scanning.

### Assessment of Dysphagia

#### Noninstrumental Assessment

We used the Schedule for Oral Motor Assessment (SOMA) to evaluate motor oral dysfunction (*12*). SOMA is a standardized discriminative assessment that quantifies oropharyngeal dysphagia in children 8–24 months of age. It is used to evaluate oral motor dysfunction and is considered one of the strongest measures for oropharyngeal dysphagia in children with neurologic disabilities (*13*). The tool categorizes children as having oral motor dysfunction or normal function on the basis of specified thresholds for each of 2 oral motor challenge categories: pureed and semisolid foods. A speech pathologist conducted an assessment using the adapted SOMA protocol (*12*).

#### Instrumental Assessment

We used 2 instrumental methods, FEES and VFSS, to evaluate all 3 phases of dysphagia; these methods are considered the reference standards for instrumental evaluation of all phases of dysphagia. FEES and VFSS are graded on an 8-point scale (the Rosenbek scale) (*14*) to assess and grade aspiration and penetration. (Aspiration is defined as passage of materials through the vocal folds, and laryngeal penetration is defined as passage of materials into the larynx, but not through the vocal folds [into the airway].)

##### FEES

FEES was performed on the infants after they underwent physical examination and clinical consultation with an otolaryngologist. FEES was performed according to the Langmore protocol (*15*) by using a 3.2-mm fiberoptic nasopharyngolaryngoscope (Machida Endoscope Co., Ltd., Tokyo, Japan). The captured images were recorded (Innova Micro-Camera MFX 10G and Halogen Light FX 180R; Innova Techinik, São Paulo, Brazil) and transferred to an Inter Core i3-2348M N3 laptop computer (Intel, Recife, Brazil). The otolaryngologist assessed the anatomy and deglutition of the infants with the help of a speech pathologist and recorded the findings for further evaluation.

During the assessment, we gave each infant the following directly in their mouth via a metered syringe: liquids (without thickener); 1 spoon thickened liquid (50 mL of liquid with 3 g of thickener); and 2 spoons food paste or puree, each with 3 g of thickener in 50 mL of liquid. We sequentially administered 1 mL, 3 mL, and 5 mL of each mixture. We used Sustap (Prolev, Abreu e Lima, Brazil) as thickener and added liquid indigo blue food dye to the preparations to obtain better visualization of the food bolus during swallowing. To minimize the risk of aspiration, we varied the sequence of administered mixtures (i.e., paste first, followed by thickened liquid and liquid) and the amount of food given, according to each infant’s clinical evaluation and information obtained from otolaryngologist’s consultation. Foods were given by a speech pathologist using a 5-mL syringe; maneuvers to facilitate swallowing were performed when needed.

##### VFSS

The digital fluoroscopy examinations were performed using a Precision GE RXi fluoroscopic radiography system (GE Healthcare, Waukesha, WI, USA). Digital images were transmitted by using a picture archiving and communication system; the images were uploaded in a computerized system and recorded in a standard DVD. The dynamic recording minimum was 3 video frames/s. Oral, pharyngeal, and cervical esophagus swallowing phases were studied with infants positioned in the lateral position. For some infants, the examination was extended on the medium and distal esophagus with infants positioned in an orthostatic laterolateral position. For this study, we added barium sulfate (Bariogel; Cristália, São Paulo, Brazil) to the testing liquids and foods to obtain better visualization during swallowing. Liquids were administered by bottle and cup, and food paste or puree (prepared as in the FEES study) was administered by spoon.

### Assessment of FEES and VFSS

We assessed 6 parameters for the diagnosis of dysphagia by FEES and VFSS: 1) premature spillage; 2) delay in initiation of swallowing; 3) residue of the bolus in the oropharynx after swallowing; 4) residue of the bolus in the hypopharynx after at least 3 swallows (i.e., saliva, secretions, or swallowed materials [contrast-enhanced in VFSS] accumulated in the valleculae, on the lateral or posterior pharyngeal walls, or in the piriform sinuses after deglutition); 5) laryngeal penetration (i.e., presence of contrast [VFSS] or food residues encroached in the airway, above the vocal folds with or without coughing [FEES]); and 6) laryngotracheal aspiration (i.e., presence of contrast-enhanced or noncontrast-enhanced materials below the vocal folds) (*16*,*17*). To quantify the severity of the last 2 parameters, laryngeal penetration and laryngotracheal aspiration, we used the 8-point Rosenbek scale (*14*), in which a score of 1 indicates absence of aspiration or penetration material in the airways; scores of 2–5 indicate degrees of penetration into the airway; and scores of 6–8 indicate degrees of aspiration. Penetration is scored as 2 or 3 if material remains above the vocal folds and as 4 or 5 if material contacts the vocal folds. Each successive score on the scale indicates a more severe sign of dysphagia than the score preceding it. Thus, a score of 8 indicates the most severe condition: aspiration of material without a reflexive or conscious attempt to expel it, which is referred to clinically as silent aspiration.

### Other Collected Data

We used a standard form to abstract individual demographic and clinical data from records, including reports by mothers of rash during pregnancy. All described investigations were conducted as part of the clinical protocol or for a clinical indication; none was conducted for research reasons alone. The study was approved by the Ethics Committee on Research (CAAE: 52803316.8.0000.5192).

## Results

**Figure 1:**
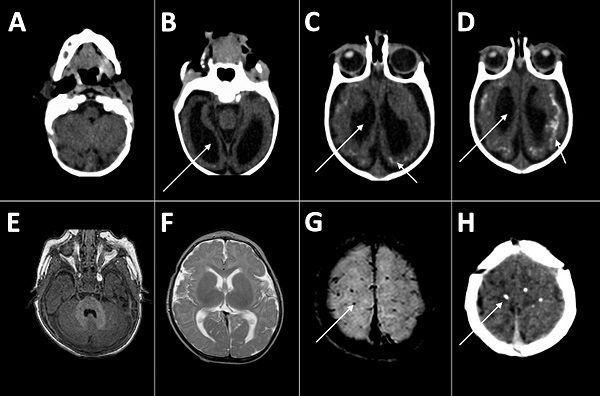
Computed tomography radiographs of the brains of 2 infants with dysphagia and microcephaly caused by congenital Zika virus infection, Brazil, 2015. A–D) Images for patient 4 show malformation of cortical development, ventriculomegaly (long arrows), and calcifications in cortical and subcortical white matter in transition between cortex and white matter (short arrows). E–H) Images for patient 6 show no malformation of cortical development or ventriculomegaly, but calcifications are visible in the cortical area (arrows).

Nine infants of various ages and with different characteristics at assessment (Technical Appendix Table 1) were referred for dysphagia investigation and received complete instrumental assessment. All infants had a degree of neurologic damage, with global developmental delays, hypertonia of the limbs, and pyramidal and extrapyramidal signs; most infants had abnormal movement of the tongue, contributing to dysphagia. The hypertonia caused abnormal posture with hyperextension of the neck in some infants. Neck hyperextension was associated with irritability, a frequent symptom in children with CZS (6/9 children in this series), and was a contributing factor to dysphagia. Only 2 of the 9 infants (patients 2 and 6) made any degree of visual contact, and only patient 6 interacted well with the environment. Patient 6 was the only infant who showed any motor development (good neck control and palmar grasping with the right hand) (Technical Appendix Table 1). Results of brain imaging for the infants were consistent with those described elsewhere for infants with CSZ microcephaly (*18*): all showed calcifications, predominantly in the cortical and subcortical region, and particularly in the border between white matter and cortex; malformations of the cortical development were present in 8 of the 9 infants (Figure 1). Only 3 of the infants had cerebellum or brainstem hypoplasia (Technical Appendix Table 2).

Onset of dysphagia was after the third month of life in 8 of the 9 infants. According to their mothers, the first symptoms of dysphagia for most of the infants were choking, cough, regurgitation, respiratory infections, and extended feeding time. All children were being fed by mouth with thickened liquids; 2 infants (patients 2 and 9) needed a nasoenteral tube after 6 months of age (Table).

**Table:** Clinical features in 9 infants with dysphagia due to congenital Zika syndrome microcephaly, Brazil, 2015

Patient no.	Age, mo, at onset of dysphagia	Main symptoms*	Respiratory infection (no.)/hospitalization (no.)	Feeding intervention	Oral feeding time >30 min†
1	4	Irritability, coughing or choking when eating or drinking, breast-feeding difficulties	No	Food thickening	No
2	4	Breast-feeding difficulties, regurgitation, weight loss	No	Nasoenteral tube (at age 6 mo)	Yes
3	3	Breast-feeding difficulties, choking when drinking	No	Food thickening	Yes
4	6	Regurgitation, coughing or choking when eating or drinking, respiratory infections	Yes (1)/Yes (>3)	Food thickening	Yes
5	2	Coughing or choking when eating or drinking	No	Food thickening	No
6	6	Regurgitation	No	Food thickening	No
7	7	Respiratory infections	Yes (2)/Yes (1)	Food thickening	Yes
8	2	Coughing or choking when eating or drinking, regurgitation, respiratory infections, weight loss	Yes (1)/Yes (1)	Food thickening	Yes
9	5	Regurgitation, coughing or choking when eating or drinking, respiratory infection	Yes (1)/Yes (1)	Nasoenteral tube (at age 11 mo)	Yes

*Reported by parent or guardian. †Before feeding interventions.

### Anatomic Assessment

Our FEES assessment of each infant’s respiratory tract showed no malformations or anatomic or functional anomalies. In each infant, the larynx was symmetrical, and mobility of the vocal cords was preserved. A common finding was an omega-shaped epiglottis, but this is common in infants and not considered pathologic.

### Abnormal Oral Phase

According to the SOMA and FEES assessments, the oral phase of swallowing was abnormal in all 9 infants. Abnormalities were characterized by premature spillage, presence of bolus residue in the oral cavity and oropharynx (except in patient 6), and marked loss of voluntary activity during the oral phase of swallowing, which is directed by the brain cortex Technical Appendix Table 3).

All 9 infants had oral motor dysfunction for pureed food, as demonstrated in the SOMA assessment. Eight infants had significant lack of function in the labial sphincter; only 1 (patient 6) was able to achieve complete labial closure. There were predominant dysfunctions during upper and lower lip activities and, at the same time, an ungraded jaw opening (ability to judge how far the mouth needs to open) while introducing the spoon into the oral cavity. Tongue protrusion beyond incisors was observed in all infants. These findings are evidence of a major impairment of the oral phase of swallowing, which leads to difficulties in oral control and premature spillage of bolus, increasing oral transit time for the bolus preparation.

One of the infants refused semisolid food, so we assessed only 8 infants. All but 1 (patient 6) of the assessed infants had oral motor dysfunction for semisolid food. Changes associated with graded jaw opening while a spoon was introduced into the oral cavity were observed in all infants, pointing to dysfunctions of jaw grading.

### Increased Risk of Aspiration in Pharyngeal Phase

**Figure 2:**
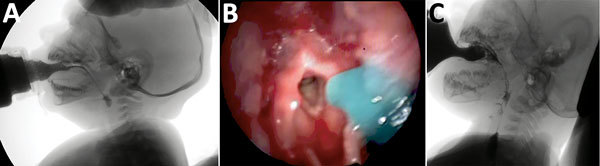
Instrumental evaluation of an infant with dysphagia and microcephaly caused by congenital Zika virus infection, Brazil, 2015. A) Videofluoroscopic swallowing study image showing a lateral view of the infant with premature spillage of liquid food (with added contrast) in the pharynx before triggering of the swallowing reflex. B) Image of the fiberoptic endoscopic evaluation of a delay in initiation of swallowing; thickened, dyed liquid is visible on the supraglottis. C) Silent aspiration, as indicated by a Rosenbek score of 8.

Eight of the 9 infants had loss of pharyngeal and laryngeal sensitivity leading to delays in initiation of the pharyngeal (or semiautomatic) swallowing phase and evidence that the swallowing reflex was triggered only when the bolus arrived on pyriform sinuses or the retrocricoid region, a delay that is associated with increased risk of aspiration before swallowing (Figure 2). FEES results showed penetration, aspiration, or both in all 9 infants; VFSS results showed these effects in 4 of the 9. FEES showed no cough reflex, characterizing silent aspiration, in 3 infants (Technical Appendix Table 3); this finding was more evident with liquid than with pureed food. After the swallowing reflex was completed, 5 of 9 infants had complete pharyngeal clearance. Those with incomplete clearance benefited from digital stimulation of swallowing, a maneuver that efficiently triggered the swallowing reflex.

### Insufficient Volume to Assess Esophageal Phase

Because of their clinical condition, 7 of the 9 infants could not swallow a sufficient volume of contrast to enable analysis of esophageal transit time. The 2 infants who could be assessed had delays in emptying the distal esophagus of test material with contrast medium.

## Discussion

Dysphagia with onset from the third month of age appears to be a common feature of CZS-associated microcephaly. In this case series, dysphagia appears to have resulted from cortical and extrapyramidal neurologic damage that led to disorganization of voluntary swallowing activity, including oral phase dysfunction with alterations in food capitation and labial closure, loss of food from the mouth, wrong positioning of the bolus, and bolus ejection. This dysfunction leads to premature spillage of bolus and increased risk of airway penetration or aspiration before the swallowing reflex is triggered, which are compatible with sensorial alterations in the oral cavity, pharynx, and larynx, leading to delays in the start of the pharyngeal phase. Liquid foods were more likely than pureed food to lead to penetration, aspiration, or both.

Few articles have been published on dysphagia in children with severe disability and nonprogressive, chronic encephalopathy; most published articles refer to dysphagia in persons with cerebral palsy. Gise et al. (*19*) described oral motor dysfunction in up to 90% of persons with cerebral palsy. Calis et al. (*20*), Fung et al. (*21*), and Santoro et al. (*22*) suggested that the severity and prevalence of dysphagia in children with cerebral palsy are strongly associated with gross motor functional capacity. van den Engel-Hoek et al. (*23*) showed the influence that dysfunction in any of the different levels of central and peripheral nervous system functions involved in initiating, coordinating, and modulating the swallowing process might have in causing dysphagia in 1 or all 3 phases of swallowing. 

Dysphagia in children with different neurologic etiologies is characterized by considerable variability. Cameron et al. (*24*) described 23 patients with symptomatic congenital cytomegalovirus infections and showed that symptomatic infections often cause a generalized encephalopathy with global functional deficits (including severe swallowing and cognitive impairments) and correlate with severity as determined by the Gross Motor Function Classification System (https://www.canchild.ca/en/resources/42-gross-motor-function-classification-system-expanded-revised-gmfcs-e-r).

The severe form of CZS with microcephaly usually causes severe encephalopathy with cerebral palsy secondary to brain injury, including involvement of cortical and subcortical areas, the basal ganglia, and, in some cases, the brainstem. Some patients with CZS may also have arthrogryposis with involvement of the lower motor neuron. All of these patterns of injuries can cause interference in any phase of swallowing. 

As of this writing, children with microcephaly resulting from CZS have only been followed for up to a maximum of 18 months after onset, but it is known that they have multiple disabilities affecting motor function, cognition, sight, and hearing. CZS is a new syndrome, and so far, there are no adequate standardized instruments for the rigorous, integrated, complete evaluation of all the deficits involved.

The discordance between results from FEES and VFSS is well described in the literature (*25*). The discordant results in this series probably resulted from the infants’ intolerance to FEES, which led to crying and irritability that, in turn, could have facilitated penetration, aspiration, or both. Although VFSS scores agreed more closely with the clinical evaluation, FEES allows a better evaluation of the pharyngeal phase of swallowing and usually can assess laryngeal sensitivity, which cannot be assessed by VFSS. In addition, FEES can be conducted as a bedside examination and is less costly than VFSS (*26*). However, information from VFSS and FEES is complementary.

To characterize dysphagia among infants in this study, we used the 2 most widely used instrumental methods: VFSS and FEES. The American Speech-Language-Hearing Association (http://www.asha.org/) considers these tests the reference standards for diagnosing dysphagia and assessing its management in order to decrease ill health and prevent death.

FEES is a dynamic test conducted in real time that provides direct visualization of the pharyngeal phase of swallowing and investigation of anatomic structures and their sensitivity. One limitation of FEES is that it requires use of nasofibroscopy, which often causes discomfort and crying in breast-feeding babies. Children with CZS microcephaly are extremely irritable, possibly because of spasms, epilepsy, or both. This was the case in 6 of the 9 infants in this series; thus, we did not feel that we could consider evaluating pharyngeal and laryngeal sensitivity in the infants.

All instrumental dysphagia evaluations require that the patients have adequate posture for testing and that, to a degree, they cooperate with the testing. Infants with CZS and microcephaly are irritable, are hyperexcited to external stimuli, and retain some primitive reflexes, so they will always be a difficult population to evaluate, in particular using FEES, which can trigger irritability and crying and interfere with the evaluation of penetration, aspiration, or both, especially in the first few months of life. There were no severe complications concerning the endoscopic procedure, suggesting that this examination is safe in this population.

The challenge with VFSS was the small volume ingested and the need to minimize exposure time to x-rays, factors that limited the usefulness of the analysis of transit. This study was based on only 9 cases; however, we expect that knowledge about dysphagia in infants with CZS and microcephaly will increase as more affected children with a wider range of disabilities are studied by instrumental assessment.

In the first few months of life, swallowing is a reflex activity, as the child feeds essentially through suction. The oral or preparatory phase, usually established at that age, is a voluntary activity that requires intact cortical function, which is absent in many children with CZS. In the infants in this series, the transfer of swallowing from a reflex activity to a complex voluntary sensorimotor process led to abnormal oral phase movements that disrupted normal swallowing after 3 months of age. The severity of the abnormal findings in the infants’ brain images is consistent with the infants’ symptoms. Compared with the other infants, patient 6, who had no congenital malformation of the cortex, had the least severe dysphagia; the only normal SOMA results; better coordination between suction, swallowing, and breath in VFSS; and clearance of food during the oral and pharyngeal phases. These findings suggest that cortical damage causes the dysfunction in the voluntary phase of swallowing.

This preliminary case report is not intended to be a full evaluation of all cases, or even a representative sample, of CZS-associated or CZS microcephaly–associated dysphagia; such an evaluation will result from the follow-up of representative cohorts. Our results are intended to inform ongoing studies and clinical management of dysphagia in children with CZS or CZS microcephaly.

In conclusion, dysphagia resulting from CZS microcephaly is severe and has onset after 3 months of age. Affected children have marked oral dysfunction, with dystonic movements of the tongue, and lack pharyngeal sensitivity, leading to risk of aspiration, in particular of liquid foods. Infants in this study were better able to swallow pureed than liquid foods; thus, we recommend that pureed or thickened food be fed to children with dysphagia caused by CZS and microcephaly. We also advise that care be taken during feeding to avoid the overextended posture characteristic of many children with CZS, a posture that facilitates aspiration. Furthermore, we recommend that, when feasible, the clinical follow-up of children with CZS should be conducted by a comprehensive and multidisciplinary team of childhood specialists in neurology, gastroenterology, speech pathology, nutrition, and otorhinolaryngology, using clinical and instrumental swallowing assessments (with FEES and VFSS) to identify different aspects of dysphagia. For clinical management, the clinical evaluation remains key.

CZS is a new syndrome, and its definition and progression are still being defined. In addition to continued research into all manifestations of CZS and the overall clinical development of affected children, we also advocate for research into whether all children born to mothers with Zika virus infection during pregnancy, regardless of the presence of microcephaly, have swallowing dysfunction when evaluated by using standardized protocols. Such a study is ongoing in Recife and will establish the prevalence, characteristics, and evolution of dysphagia as well as the possibility of onset of any abnormalities of the intestinal tract.

Technical Appendix Characteristics and computed tomography scan and dysphagia assessment results for infants with microcephaly caused by congenital Zika virus infection, Brazil, 2015.
