# Supplementary material for: Characteristics of Dysphagia in Infants with Microcephaly Caused by Congenital Zika Virus Infection, Brazil, 2015
Source: Emerg Infect Dis. 2017 Aug;23(8):1253–9. doi: 10.3201/eid2308.170354 (PMC5547788; doi:10.3201/eid2308.170354)
Supplement: Technical Appendix — Characteristics and computed tomography scan and dysphagia assessment results for infants with microcephaly caused by congenital Zika virus infection, Brazil, 2015. [file 17-0354-Techapp-s1.pdf]

Article DOI: https://doi.org/10.3201/eid2308.170354

Characteristics of Dysphagia in Infants with Microcephaly Caused by Congenital Zika Virus Infection, Brazil, 2015

Technical Appendix

Technical Appendix Table 1. Characteristics of the children including neurological evaluation.

No	SEX	Gestational age, weeks	Head circumference, CM	Birth weight for gestational age	Microcephallus, according to INTERGROWTH 21ST (*1*)	Pregnancy rash, month	Visual fixation and pursuit	Ocular motility	Head control	Social smile	Sitting without support	Grasp	Asymmetric tonic neck reflex	Limbs hypertonia with pyramidal and extrapyramidal signs	Irritability	Epilepsy
1	F	36	27	Appropriate	3SD	4	No	Nistagmus	No	No	No	Grasping reflex	Yes	Yes	No	Yes
2	M	38	28.5	Appropriate	3SD	2	Yes	Nistagmus	No	Yes	No	Grasping reflex	Yes	Yes	Yes	Yes
3	F	39	28.5	Appropriate	3SD	2	No	Nistagmus	No	No	No	Grasping reflex	Yes	Yes	Yes	Yes
4	F	37	30	Appropriate	2SD	3	No	Strabismus	No	No	No	Grasping reflex	Yes	Yes	Yes	Yes
5	M	38	28	Small	3SD	1	No	Strabismus	No	No	No	Grasping reflex	Yes	Yes	No	Yes
6	M	39	31	Appropriate	2SD	2	Yes	Normal	Yes	No	No	Palmar grasp, pick-up large objects	No	Yes	No	No
7	M	39	26	Small	3SD	3	No	Strabismus	No	No	No	Grasping reflex	Yes	Yes	Yes	Yes
8	F	40	28.5	Appropriate	3SD	7	Yes	Strabismus	No	No	No	Grasping reflex	Yes	Yes	Yes	Yes
9	F	39	27	Small	3SD	6	Yes	Normal	No	No	No	Grasping reflex	Yes	Yes	Yes	Yes

Technical Appendix Table 2. Computerized tomography scan findings

	CT scan finding									
Patient no.	Decreased brain volume	Cerebellum or brainstem hypoplasia	Ventriculomegaly	Malformations of cortical development	Cortical and subcortical white mater calcifications	Basal ganglia calcifications	Cerebellum calcifications	Periventricular calcifications	Brainstem calcifications	Enlarged cisterna magna
1	Yes	Yes	Yes	Yes	Yes	Yes	No	No	No	No
2	Yes	No	Yes	Yes	Yes	Yes	No	Yes	No	Yes
3	Yes	Yes	Yes	Yes	Yes	Yes	No	No	No	Yes
4	Yes	No	Yes	Yes	Yes	No	No	No	No	Yes
5	Yes	Yes	Yes	Yes	Yes	Yes	No	Yes	No	Yes
6	Yes	No	No	No	Yes	No	No	No	No	No
7	Yes	No	Yes	Yes	Yes	Yes	No	No	No	Yes
8	Yes	No	Yes	Yes	Yes	No	No	No	No	No
9	Yes	No	Yes	Yes	Yes	No	No	No	No	No

Technical Appendix Table 3. Dysphagia: assessment results using FEES, VFSS and SOMA in nine children with CZS microcephaly

		FEES/VFSS							SOMA	
Patient no.	Age, mo, FEES/VFSS	Premature spillage	Delay in initiation of swallowing	Residue of the bolus in the oropharynx after swallowing	Residue of the bolus in the hypopharynx after swallowing	Laryngeal penetration	Laryngotracheal aspiration	Rosenbeck score*	Oral motor dysfunction, puree	Oral motor dysfunction, semi-solid
1	09/10	+/+	+/+	+/+	-/-	+/-	+/-	8/1	+	+
2	08/10	+/+	+/-	+/+	+/+	+/+	-/+	5/8	+	+
3	09/10	+/+	+/+	+/+	+/+	+/+	+/+	7/8	+	+
4	09/09	+/+	+/+	+/+	-/-	+/-	+/-	7/1	+	+
5	16/11	+/+	+/+	+/+	+/+	+/+	+/+	7/6	+	+
6	12/11	+/+	+/+	-/-	-/-	+/-	-/-	5/1	+	-
7	09/09	+/+	+/+	+/+	-/-	+/-	+/-	7/1	+	†
8	09/09	+/+	+/+	+/+	+/-	+/-	-/-	5/1	+	+
9	08/12	+/+	-/+	+/+	-/-	-/+	-/-	1/5	+	+

FEES: Fiberoptic endoscopic evaluation of swallowing; VFSS: Videofluoroscopic Swallowing Study; SOMA: Schedule for Oral Motor Assessment.
*Rosenbek score (*2*): 1- Material does not enter the airway; 2- Laryngeal penetration (material enters the airway, remains above the vocal folds, and is ejected from the airway; 3- Material enters the airway, remains above the vocal folds, and is not ejected from the airway; 4- Material enters the airway, contacts the vocal folds, and is ejected from the airway; 5- Material enters the airway, contacts the vocal folds, and is not ejected from the airway); 6-Laryngotracheal aspiration (material enters the airway, passes below the vocal folds and is ejected into the larynx or out of the airway; 7- Material enters the airway, passes below the vocal folds, and is not ejected from the trachea despite effort, 8-Material enters the airway, passes below the vocal folds, and no effort is made to eject).
†The child refused semi-solid food.

References

1. Villar J, Cheikh Ismail L, Victora CG, Ohuma EO, Bertino E, Altman DG, et al.; International Fetal and Newborn Growth Consortium for the 21st Century (INTERGROWTH-21st). International standards for newborn weight, length, and head circumference by gestational age and sex: the Newborn Cross-Sectional Study of the INTERGROWTH-21st Project. Lancet. 2014;384:857–68. PubMed http://dx.doi.org/10.1016/S0140-6736(14)60932-6

2. Rosenbek JC, Robbins JA, Roecker EB, Coyle JL, Wood JL. A penetration–aspiration scale. Dysphagia. 1996;11:93–8. PubMed http://dx.doi.org/10.1007/BF00417897
